# Effects of oxycodone hydrochloride sustained-release tablets and morphine hydrochloride sustained-release tablets on peripheral blood T cell levels in advanced lung squamous cell carcinoma with moderate to severe cancer pain

**DOI:** 10.12669/pjms.39.5.6772

**Published:** 2023

**Authors:** Juan Yan, Zheng-zheng Xie, Na Ma, Mao-dong Zheng, Ting-ting Qiao, Yu Wang

**Affiliations:** 1 Juan Yan, Department of Pharmacy, The First Affiliated Hospital of Hebei North University, Zhangjiakou, Hebei,075000, China; 2 Zheng-zheng Xie Department of Pharmacy, Beijing Shijitan Hospital, Beijing,100038, China; 3 Na Ma Department of Pharmacy, Taian City Central Hospital, Taian, Shandong,271400, China; 4 Mao-dong Zheng, Department of Pharmacy, The First Affiliated Hospital of Hebei North University, Zhangjiakou, Hebei,075000, China; 5 Ting-ting Qiao, Department of Pharmacy, The First Affiliated Hospital of Hebei North University, Zhangjiakou, Hebei,075000, China; 6 Yu Wang, Department of Pharmacy, The First Affiliated Hospital of Hebei North University, Zhangjiakou, Hebei,075000, China

**Keywords:** Lung Squamous Cell Carcinoma, Morphine Hydrochloride Sustained-Release Tablet, Cancer Pain, Oxycodone Hydrochloride Sustained-Release Tablet

## Abstract

**Objective:**

To investigate the effects of morphine hydrochloride sustained-release tablets and oxycodone hydrochloride sustained-release tablets on T-cell levels in advanced lung squamous cell carcinoma(LUSC) with moderate to severe cancer pain.

**Methods:**

A retrospective study was used, ninety-eight patients who were admitted to The First Affiliated Hospital of Hebei North University for treatment of advanced LUSC with moderate to severe cancer pain between January 2021 and December 2021 were randomized into two groups(n=49 each) using the sealed envelope system. The reference group was treated with morphine hydrochloride sustained-release tablets, while the experimental group received oxycodone hydrochloride sustained-release tablets to compare pain relief rates(PRRs), levels of T cells, pain intensity, et al. Blood samples were collected for lymphocyte levels by flow cytometry.

**Results:**

The experimental group had significantly higher level than the reference group(*P*<0.05). Before administration, the two groups did not differ greatly in levels of T-cell subsets or pain scores on the visual analog scale(*P*>0.05, respectively). At 15 days of administration, the Treg level in the experimental group was higher than in the reference group; T helper 17 and 22 cells were reduced in both groups, and the decrease was more pronounced in the experimental group. At seven and 15 days of administration, the experimental group had a VAS score significantly lower than the reference group(*P*<0.05). The total adverse reaction rate was significantly lower in the experimental group as compared with the reference group(*P*<0.05).

**Conclusions:**

Oxycodone hydrochloride sustained-release tablets demonstrate desirable efficacy and safety in advanced LUSC with moderate to severe cancer pain by modulating T-cells in the body and improving the PRR.

## INTRODUCTION

Lung cancer is a common clinical malignancy and the leading cause of cancer death in China.^1^,^2^ Pain, as the most frequently occurring chronic symptom in cancer patients, is characterized by the primary clinical manifestation as generalized, intense pain, and evident psychological and autonomic nervous system (ANS) abnormalities.^3^,^4^ Rational use of drugs is considered one of the effective approaches to cancer pain management. Despite the established efficacy and wide application among patients with moderate to severe cancer pain, morphine hydrochloride sustained-release tablets can cause serious side effects and even addiction.^5^

Oxycodone hydrochloride sustained-release tablets represent a type of potent, semi-synthetic analgesic agent that helps relieve pain and improve prognosis.^6^ However, it is important to further investigate the mechanism of oxycodone hydrochloride sustained-release tablets as a modulator of T-cell subsets in advanced LUSC patients with moderate to severe cancer pain. Therefore, this study enrolled 98 advanced LUSC patients with moderate to severe cancer pain to analyze and compare the application value of these two analgesic agents.

## METHODS

A retrospective study was used in this study during January and December 2021, 98 patients who visited The First Affiliated Hospital of Hebei North University for treatment of advanced LUSC with moderate to severe cancer pain were included in this study were randomized into two groups (n=49 each) using the sealed envelope system.

### Ethical Approval:

The study was approved by the Institutional Ethics Committee of Baoding NO.1 Central Hospital on August 4^th^, 2020 (No.:K2020310), and written informed consent was obtained from all participants.

### Inclusion criteria:

- Patients met the diagnosis criteria for moderate to severe cancer pain;^7^
- Acted compliantly in tests throughout the treatment course;
- Had an estimated survival >two months.

### Exclusion criteria:

- Patients had a psychiatric history;
- Patients missed doses frequently or discontinued medication without authorization;
- Patients were concurrently diagnosed with any other primary tumor;

Withdrew early from the study or were lost to follow-up, and/or 5) were allergic to either analgesic agent studied. The patients were randomly assigned to two groups (n =49 each) which showed a high degree of comparability in baseline data (all *P* >0.05).

The reference group was administered morphine hydrochloride sustained-release tablets (G.Y.Zh.Z. H63020014, 5mg, Qinghai Pharmaceutical), starting from 10 mg/12 hour for 15 days, and dose adjustment was required based on individual patient evaluation. The experimental group was given oxycodone hydrochloride sustained-release tablets (G.Y.Zh.Z. J20140126, 10mg, Mundipharma (China) Pharmaceutical) for 15 days; with an initial dose of 10 mg/12 h, incremental dosing should be applied according to patient’s condition if inadequate pain relief was provided throughout a 12-hour interval. Simple exercises were instructed during the treatment course.

### Outcome measures:

***Pain relief rate (PRR)^7^:*** Treatment outcomes were classified as complete response (CR), partial response (PR), mild pain (MP), and no response (NR) (CR: disappearance of almost all painful sensations and evident remission of inflammatory symptoms; PR: remarkable pain relief without sleep disturbance; MP: disturbed sleep quality despite some alleviation after treatment; NR: no observable pain relief or improvement in sleep quality).

***T-cell levels:*** Peripheral blood samples (5 mL each) were collected from both groups before and after the 15-days treatment course. Peripheral blood mononuclear cells were immediately sent to the laboratory for isolation and stored at 4°C. The NovoCyte D2041R flow cytometer system (ACEA Biosciences) was employed to determine the levels of regulatory T (Treg), T helper 22 (Th22), and T helper 17 (Th17) cells.

***Pain intensity:*** The visual analogue scale (VAS)^8^ was applied to measure pain intensity before and at 7 and 15 days of treatment, with a higher score indicative of worse pain.

***Adverse reactions:*** Occurrences of adverse reactions such as constipation, vomiting, and skin itching were documented throughout the treatment course.

### Statistical analysis:

Data analysis was conducted using SPSS23.0. Measurement data were expressed in the form of (*X̅*±*S*) and examined by the t-test. Quantitative data were represented as “n(%)” and analyzed by the chi-square test. The significant level was set at *P* <0.05.

## RESULTS

PRR was 95.92% in the experimental group (oxycodone hydrochloride sustained-release tablets) and 83.67% in the reference group (morphine hydrochloride sustained-release tablets), with the difference suggesting statistical significance (*P* <0.05). Table-II.

**Table-I:** Intergroup comparison of clinical data.

Group	n	Sex (n/n)	Age (χ̅±S, yr)	Course of Disease (χ̅±S, yr)

		M/F		
Experimental group	49	27/22	50.51±5.46	6.72±1.21
Reference group	49	26/23	50.39±5.52	6.80±1.24
Statistic		0.041	0.108	0.323
P-value		0.839	0.914	0.747

**Table-II:** Intergroup comparison of pain relief rate (PRR) [n(%)].

Group	n	CR	PR	MP	NR	PRR
Experimental group	49	18(36.73)	23(46.94)	6(12.24)	2(4.08)	47(95.92)
Reference group	49	12(24.49)	20(40.82)	9(18.37)	8(16.33)	41(83.67)
*χ2*	-					4.009
*P*	-					0.045

Before administration, no statistically significant difference was observed between the two groups in T-cell levels (*P* >0.05, respectively). At 15 d of administration, the Treg level in peripheral blood was elevated in both groups, and the experimental group (oxycodone hydrochloride sustained-release tablets) displayed a Treg level higher than that of the reference group (morphine hydrochloride sustained-release tablets); Th17 and Th22 cells were reduced in the two groups, and the levels of both were significantly lower in the experimental group than in the reference group (*P* <0.05, respectively). Table-III.

**Table-III:** Intergroup comparison of T-cell levels (*χ̅*±*S*, n =49, %).

Group	Treg		Th17		Th22	

	Before Administration	After Administration	Before Administration	After Administration	Before Administration	After Administration
Experimental group	1.51±0.28	5.02±1.08*	3.09±0.62	0.96±0.16*	2.23±0.59	0.65±0.12*
Reference group	1.55±0.30	3.84±0.57*	3.15±0.67	1.83±0.28*	2.28±0.56	1.35±0.24*
*t*	0.682	6.764	0.460	18.884	0.430	18.261
*P*	0.497	<0.001	0.647	<0.001	0.668	<0.001

***Note:***

* P <0.05 compared with the pre-administration levels.

Before administration, the two groups did not differ greatly in the VAS score (*P* >0.05). At seven and 15 d of administration, both groups scored lower on the VAS as compared with the pre-administration performance, and the experimental group (oxycodone hydrochloride sustained-release tablets) had a lower VAS score than that of the reference group (morphine hydrochloride sustained-release tablets) (*P* <0.05). Table-IV.

**Table-IV:** Intergroup comparison of pain intensity (*χ̅*±*S*, pt).

Group	n	Before Administration	At 7 d of Administration	At 15 d of Administration
Experimental group	49	6.12±1.25	2.07±0.40*	1.15±0.23*
Reference group	49	6.06±1.27	3.11±0.56*	1.98±0.30*
*t*	-	0.236	10.579	15.370
*P*	-	0.814	<0.001	<0.001

***Note:***

* P <0.05 compared with the pre-administration levels.

In the experimental group (n=49), there was zero (0.00%) constipation case, 1 (2.04%) vomiting, and 1 (2.04%) skin itching, making up a total adverse reaction rate (ADR) of 4.08% (2/49). As to the reference group, 2 (4.08%) reported constipation, 4 (8.16%) experienced vomiting, and 2 (4.08%) developed skin itching symptoms, representing a total ADR of 16.33% (8/49). The total ADR was lower in the experimental group as compared with the reference group (*P* <0.05).

## DISCUSSION

There was evidence that^9^ oxycodone hydrochloride sustained-release tablets could alleviate severe cancer pain. In this study, the PRR in the experimental group was 95.92%, significantly higher than 83.67% in the reference group. Before administration, the VAS score was not significantly different between the two groups (*P* >0.05). At seven and 15 days of administration, the VAS score declined in both groups, and the experimental group (oxycodone hydrochloride sustained-release tablets) scored lower than the reference group (morphine hydrochloride sustained-release tablets) (*P* <0.05), demonstrating the pain-relieving benefits of oxycodone hydrochloride sustained-release tablets for advanced LUSC patients with moderate to severe cancer pain. Through analysis, oxycodone hydrochloride sustained-release tablets might exert analgesic effects to achieve pain relief.^10^,^11^ The pathogenesis of advanced LUSC with moderate to severe cancer pain is strongly associated with immune system problems, typically T-cell disorders.

Lung cancer poses a grave threat to the global population with the highest incidence and mortality rates among all cancers. As a common clinical type of lung cancer, LUSC is characterized by insidious lesions and the lack of typical symptoms in the initial stage. Most LUSC cases were classified as advanced stage at diagnosis, which can cause intense compression of local tissues, including nerve fibers with free endings sensitive to painful stimuli, and result in severe pain.^12^,^13^ Since the pathogenesis of LUSC with moderate to severe cancer pain has not been fully understood, pain-relieving medicines remain the clinical mainstay of cancer pain management.

Morphine hydrochloride sustained-release tablets are formulated with the active ingredient morphine - a mu-opioid receptor agonist - and demonstrate potent analgesic activity by acting directly on the central nervous system. Although the analgesic agent provides up to 12 hour of relief with every dose, it entails potential risks of tolerance, addiction, and dependence with long-term use.^14^,^15^ Oxycodone hydrochloride sustained-release tablets are a dual-release formulation of mu-opioid receptor agonist that shows an apparent biphasic in vitro dissolution profile, with an initial release within the first hour of administration accounting for 38% of the dose, and an extended-release fraction amounting to 62% of the dose.

This powerful formulation can relieve pain for up to 12 hours, with high oral bioavailability (>60%) and an analgesic potency 1.5- to 2-fold greater than morphine hydrochloride sustained-release tablets. However, oxycodone hydrochloride sustained-release tablets can induce respiratory depression and other side effects.^16^,^17^

Advanced LUSC with moderate to severe cancer pain can induce disorders of T-cell and cytokine secretion, with a higher Th17/Th22 ratio suggesting severer immune dysfunction. Moreover, this study also showed that before administration, the two groups did not differ greatly in T-cell levels (*P* >0.05, respectively); at 15 days after administration, an elevated Treg level was observed in the peripheral blood samples of both groups, and the experimental group (oxycodone hydrochloride sustained-release tablets) had a higher Treg level as compared with the reference group (morphine hydrochloride sustained-release tablets); peripheral blood levels of Th17 and Th22 cells were decreased in both groups and were significantly lower in the experimental group than in the reference group (*P* <0.05, respectively), demonstrating the modulatory effects of oxycodone hydrochloride sustained-release tablets on the peripheral blood levels of such T cells in advanced LUSC patients with moderate to severe cancer pain.

This might be explained by the vigorous tissue-penetrating delivery of oxycodone hydrochloride to modulate T-cell subsets and improve immune function by activating granulocytes, phagocytes, and monocytes with anti-infective activity.^18^,^20^ The analysis of adverse reactions suggested that oxycodone hydrochloride sustained-release tablets had a good safety profile as adverse reactions occurred less frequently in the experimental group (oxycodone hydrochloride sustained-release tablets) as compared with the reference group (morphine hydrochloride sustained-release tablets) (*P*<0.05).

### Limitations of this study

It includes a modest sample size that implies a potential for sampling errors. The conclusions should be further validated by increasing the sample size.

## CONCLUSIONS

Oxycodone hydrochloride sustained-release tablets are are safe and effective and canbe used for treatment of advanced LUSC patients with moderate to severe cancer pain to modulate T-cell levels and improve the PRR.

### Authors’ Contributions:

**JY and ZX:** Designed this study, prepared this manuscript, are responsible and accountable for the accuracy and integrity of the work.**NM and** **MZ:** Collected and analyzed clinical data.**TQ and** **YW:** Data analysis**,** Significantly revised this manuscript.
